# Zhenqing recipe attenuates non-alcoholic fatty liver disease by regulating the SIK1/CRTC2 signaling in experimental diabetic rats

**DOI:** 10.1186/s12906-019-2811-2

**Published:** 2020-01-31

**Authors:** Daofei Song, Lei Yin, Chang Wang, Xiuying Wen

**Affiliations:** 1 Department of Endocrinology, Liyuan Hospital, Tongji Medical College, Huazhong University of Science and Technology, Wuhan, People’s Republic of China; 2 Department of Endocrinology, Hubei Provincial Hospital of Integrated Chinese and Western Medicine, Wuhan, People’s Republic of China; 3 Department of Traditional Chinese Medicine and Endocrinology, Liyuan Hospital, Tongji Medical College, Huazhong University of Science and Technology, 39 Lake Avenue, Wuhan, Hubei 430077 People’s Republic of China

**Keywords:** Zhenqing recipe, High-fat diet, Streptozotocin, Rats, Diabetes, Nonalcoholic fatty liver disease, Gluconeogenesis, Salt-induced kinase 1

## Abstract

**Background:**

As a compound Chinese medicine, Zhenqing Recipe (ZQR) has been shown to ameliorate hyperglycemia, hyperlipidemia, fatty liver and insulin resistance in patients with diabetes and diabetic rats. In this paper, we further examined the effect of ZQR on diabetes complicated by non-alcoholic fatty liver disease (NAFLD) and the underlying molecular mechanisms.

**Methods:**

Diabetic rats with NAFLD were developed by a high-fat diet (HFD) with low-dose streptozotocin (STZ) injection for 4 weeks. These rats were randomly separated into the diabetic model (DM), ZQR, metformin (Met), adenovirus expressing-salt-induced kinase 1 (Ad-SIK1) and adenovirus labeled with green fluorescent protein (Ad-GFP) groups. The effects on hepatic expression of gluconeogenic genes, glycolipid metabolism and pathological changes were subsequently detected.

**Results:**

Serum glucose, triglycerides (TG), total cholesterol (TC) and hepatic TG were reduced in the ZQR group. The histopathological and immunohistochemical changes in the liver and pancreas in the ZQR group were significantly alleviated. The decrease of SIK1 expression was observed in the liver of diabetic rats induced by HFD and STZ. SIK1 overexpression in the liver relieved hyperglycemia, hyperlipidemia and fatty liver. Both the mRNA and protein levels of CREB-regulated transcription co-activator 2 (CRTC2), phosphoenolpyruvate carboxykinase (PEPCK) and glucose-6-phosphatase (G6Pase) in the liver were drastically reduced, whereas those of SIK1 were markedly increased in the ZQR group compared to levels in the DM group. Compared with the DM group, Ser577 phosphorylation of SIK1 was obviously reduced in the liver, while T182 phosphorylation of SIK1 and S171 phosphorylation of CRTC2 were evidently increased in the Ad-SIK1, Met and ZQR groups.

**Conclusions:**

ZQR ameliorates hepatic gluconeogenesis and lipid storage in diabetic rats induced by HFD and STZ by activating the SIK1/CRTC2 signaling pathway. Upregulating hepatic SIK1 by ZQR may represent an efficient strategy for treating diabetes with NAFLD.

## Background

Type 2 diabetes mellitus (T2DM) has three main characteristics: hyperglycemia, insulin resistance, and impaired islet cell function and is the most common type of diabetes worldwide [1]. T2DM causes not only hyperglycemia but also hyperlipidemia, diabetic nephropathy, and NAFLD [2]. Among these complications, NAFLD is one of the most common symptoms, characterized by excessive lipid accumulation in the form of TG, which is highly correlated with several metabolic diseases, such as hyperlipidemia, insulin resistance (IR) and T2DM [3, 4]. Furthermore, the prevalence of NAFLD in obese and diabetic populations is far higher than that in normal populations, which often leads to severe diseases such as steatosis, non-alcoholic steatohepatitis, fibrosis and cirrhosis [5]. Therefore, attenuating this complication of T2DM is equally as important as decreasing blood glucose levels in long-term therapies.

Salt-induced kinase 1 (SIK1) plays an important role in glycolipid metabolism [6, 7]. As one of the AMP-activated protein kinases family members, SIK1 significantly suppress hepatic glucogenesis and lipogenesis [8–11]. It has been reported that liver kinase B1 (LKB1) phosphorylates SIK1-Thr182, which is indispensable for activating the SIK1 activity, resulting in increased kinase activity of SIK1 [12, 13], while LKB1 is the upstream kinase of AMPK. As described in detail previously [8, 14], SIK1 translocates to the cytoplasm and loses its inhibitory features after Ser-577 is phosphorylated by protein kinase A (PKA) due to adrenocorticotropic hormone (ACTH) treatment. SIK1 has multiple physiological functions such as the regulation of hepatic gluconeogenesis and lipogenesis, which is closely related to diabetes mellitus and NAFLD [6, 7, 14].

The liver plays a vital role in regulating glycolipid metabolism. Glucose production in the liver primarily originates from gluconeogenesis [15]. Hepatic gluconeogenesis is regulated by modulation of the expression of PEPCK and G-6-Pase [16]. The cAMP response element binding protein (CREB) and its co-activator, CRTC2, play an important role in regulating gluconeogenesis in the liver. CREB induces hepatic gluconeogenesis and then activates gluconeogenic enzymes such as PEPCK and G6Pase [17–19]. CRTC2 regulates glucose production via CREB. Moreover, CRTC2 contributes to the development of hepatic insulin resistance and steatosis through its effects on hepatic gluconeogenesis [20, 21]. Intriguingly, a previous report illustrated that SIK1 phosphorylates CRTC2 and thereby inhibits downstream gluconeogenic genes, including PEPCK and G6Pase [22]. Therefore, the SIK1/CRTC2 signaling pathway may be a target for treating T2DM. Diabetes causes disturbance of glucose and lipid metabolism. Therefore, identifying strategies to activate SIK1 may regulate the disorder of glycolipid metabolism in patients with diabetes with NAFLD through the SIK1/CRTC2 signaling pathway.

Presently, no medication is approved for NAFLD. Several medicines have been suggested, but none have shown significant effects on liver damage [23]. Weight loss and lifestyle modification have usually been applied for the treatment of NAFLD but are often difficult to maintain for patients. Therefore, there is an urgent need to identify medications targeted at increased hepatic fat that are safe for long-term administration. ZQR is a Chinese herbal formula. Clinical observations indicated that ZQR decreased blood sugar, serum triglyceride and serum cholesterol in patients with T2DM [24]. Our previous studies indicated that ZQR reduced blood sugar and lipids, but also significantly ameliorated fatty liver in diabetic rats [25–27]. Recently, our research showed that ZQR significantly decreased fasting blood glucose (FBG), serum TG, serum TC and liver TG contents, while the hepatic expression of SIK1 was obviously upregulated by ZQR treatment in NAFLD rats with T2DM [28]. In this study, we further investigated whether ZQR inhibits hepatic gluconeogenesis and lipid storage, and whether this effect is connected to the SIK1/CRTC2 pathway in diabetic rats induced by HFD and STZ.

## Methods

### Preparation of ZQR extract

ZQR consists of *Ligustrum lucidum* W.T.Aiton, *Eclipta prostrata* Lour and *Dioscorea oppositifolia* L.. All herbal drugs were purchased from Tianji Traditional Chinese Herbal Company (Wuhan, China). *Ligustrum lucidum* W.T.Aiton (71.76 g), *Eclipta prostrata* Lour (71.76 g) and *Dioscorea oppositifolia* L. (89.7 g) were mixed and boiled in water (1:10, w/v) for 2 h, and then another 2-h boil was carried out. Subsequently, the solution obtained was concentrated to 0.8 g/ml. The extract solution was deposited at 4 °C until use. The mixing ratio of extracts was based on dosage ratios from previous in vitro and in vivo studies. All voucher specimens were left in the Department of Integrated Chinese and Western Medicine, Liyuan Hospital, Tongji Medical College, HUST (China) with numbers 201,804,001, 201,804,002, 201,804,003, 201,804,004, 201,804,005, 201,804,006, 201,804,007, 201,804,008, 201,804,009 and 201,804,010.

### Reagents

Antibodies applied in this work were against SIK1 from Novus (USA); against G6Pase and CRTC2 (S171) from Abcam (UK); against CRTC2, SIK1 (S577), and SIK1 (T182) from ProteinTech (USA); against PEPCK andβ-actin from Cell Signaling Technology (USA); Goat anti-rabbit IgG from Bioworld Technology (USA). Chemicals used in this study were Goldview DNA dye and DNA Marker I from Tiangenshengwu Technology Co., Ltd. (China); Metformin from Sino-American Shanghai Squibb Pharmaceuticals Co., Ltd. (China).

### Adenovirus vector and treatment

SIK1 cDNA (NM_021693) was obtained from the cDNA library of Genechem (Shanghai, China). The 2337 base pair PCR product was cloned into a linearised adenovirus plasmid GV314 (Genechem) with T4 DNA ligase and transfected into competent *Escherichia coli* cells. Positive clones were selected by ampicillin resistance and then sequenced by ABI3730 sequencing analysis (Invitrogen, Shanghai, China). The SIK1 overexpression adenovirus (Ad-SIK1) was packaged in HEK293T cells and purified with an Adeno-X™ Virus Purification Kit (BD Biosciences, San Jose, CA, USA). The endpoint dilution method was used to determine the viral titre. Adenovirus particles containing CMV-MCS-3FLAG-SV40-EGFP (Ad-GFP; purchased from Genechem) served as a negative control. The obtained adenovirus was stored at − 80 °C.

Diabetic rats were injected with adenovirus at an optimized dose of 5 × 10^9^ PFU in 50 μl via the tail vein once a week for 8 weeks. Additionally, the rats of the control and model groups were injected with physiological saline at the same dosage by tail vein.

### Animals and drug administration

Sixty male Wistar rats, three to four-weeks-old, weighing 83.2 ± 9.7 g, were purchased from Huafukang Technology Co., Ltd. (China). All procedures were approved by the Animal Ethics Committee of Tongji Medical College, Huazhong University of Science and Technology (IACUC Number: 822).

Animals were housed in a specific pathogen free (SPF) room (22 °C ± 3 °C, 50% ± 5% humidity, and a 12/12-h circadian rhythm) with free access to water and diet. During housing, animals were monitored once a day for health status. No adverse events were observed. After 1 week of adaptive feeding, the rats were randomly divided into two groups. One group was given a normal diet (containing 13.68% fat, 64.44% carbohydrate, and 21.88% protein), and the other group was given a HFD (containing 52.5% standard laboratory rat chow, 20% lard, 10% sugar, 10% imported fish meal, 5% egg yolk power, 2% cholesterol, and 0.5% bile salts) [26]. After 4 weeks, rats on the HFD were given intraperitoneal injections of STZ (36 mg/kg) dissolved in citrate buffer (pH 4.5), whereas the remaining rats were given intraperitoneal injections of the same volume of citrate buffer. Diabetes was identified when the serum glucose level of rats was greater than or equal to 11.1 mmol/L 72 h after STZ injection. The diabetic rats were randomized into the following five groups (*n* = 8 per group): DM group, ZQR group, metformin (Met) group, Ad-Sik1 group, and Ad-GFP group.

In the ZQR and Met groups, rats were given oral ZQR extract (3.78 g/kg/bw/day) and metformin suspension (150 mg/kg/bw/day) by gavage, respectively. Rats were given the same amount of distilled water by gavage daily in the DM and normal control groups. The Ad-Sik1 and Ad-GFP groups were given a 0.05-ml injection of Ad-SIK1 (1 × 10^11^ PFU/ml) and Ad-GFP (1 × 10^11^ PFU/ml) via the tail vein, respectively. Oral administration was carried out every day for 12 weeks. The normal control group and model group were given the same amount of distilled water every day. The dosage of intragastric administration was adjusted based on the weekly body weight of rats. Our ARRIVE (Animal Research: Reporting of In Vivo Experiments) guidelines were used for reporting the study and the checklist is added as Additional file 1.

Pentobarbital sodium and cervical dislocation euthanasia were performed to decrease animal suffering in the course of the experiment. After a 12-week treatment, the rats were weighed and anesthetized deeply with intraperitoneal pentobarbital sodium (30 mg/kg, Merck, USA). Subsequently, blood samples were obtained from the ventral aorta and then were centrifuged before the serum was separated and stored at − 80 °C. Rats were euthanized by cervical dislocation. The liver and pancreas were removed and weighed. Part of the liver and part of the pancreas were fixed in 4% paraformaldehyde.

### Biochemical assays

Blood glucose levels were examined using glucose oxidase kits (Ruiyuan Biotechnology, China). The serum TG and TC were measured using commercial reagents (Randox, UK). Blood insulin levels were measured by an enzyme-linked immunosorbent assay (ELISA) kit (ELK Biotechnology, China). Hepatic triglyceride was measured using commercial reagent (Jiancheng Bioengineering Institute, China).

### Histological staining

Liver and pancreas tissues were divided into 4 μm sections through formalin fixation and paraffin embedding. Next, the gross morphology of these tissues was examined by hematoxylin and eosin staining (HE). Additionally, the liver sections were determined by Oil red O staining.

### Immunohistochemistry analysis

The liver and pancreas tissues were fixed with 4% paraformaldehyde, embedded in paraffin and sectioned. Rabbit polyclonal SIK1 antibody, rabbit polyclonal CRTC2 antibody, rabbit polyclonal PEPCK antibody and rabbit polyclonal G6Pase antibody as well as rabbit polyclonal insulin antibody were used as the primary antibodies. After dewaxing with dimethylbenzene, alcohol gradient and antigen retrieval, the liver and pancreas sections were processed with 3% H_2_O_2_ to quench endogenous peroxidase, and then incubated with 10% goat serum for 20 min. The sections were treated with primary antibodies (1:100). Finally, the sections were observed by light microscopy. The pictures were analyzed with Image Pro Plus 6.0 software (USA) to calculate the integrated optical density (IOD), which was measured as previously described [29].

### RT-PCR

RNA in liver tissues was separated by RNAiso Plus (Takara, Japan) and then subjected to RT-PCR as previously described [14, 30]. The forward and reverse primers were designed and produced by Shenggong Technology Co., Ltd. (China); After electrophoresis, the obtained PCR products were divided by agarose gel, and the mRNA expression was measured using a digital recorder (China). The primer sequences are listed in Additional file 2.

### Western blot analysis

Total protein from rat liver lysates were extracted, isolated on 10 or 12% SDS-PAGE gels, and transferred to nitrocellulose membranes. Next, the membranes were blocked with 5% nonfat milk (room temperature, 1 h) dissolved in Tris-buffered saline and Tween 20 (TBST), and then were overnight incubated with primary antibodies at 4 °C. After 3 washes in TBST, the membranes were incubated for 30 min at room temperature. Immunoreactive proteins were examined using ECL reagent and normalized against β-actin with Image J software (National Institutes of Health, USA).

### Statistical analysis

The results were presented as the mean ± SD and were analyzed with GraphPad Prism 5.0 software (San Diego, USA). All data were assayed with one-way ANOVA or two-way ANOVA. *P < 0.05; **P < 0.01; ***P < 0.001. *P* < 0.05 was considered statistically significant.

## Results

### Effect of ZQR on body and liver weight in diabetic rats

The results are expressed as the mean ± SD. ^*^P < 0.05, ^**^P < 0.01, ^***^ *P* < 0.001 versus control guoup. ^#^*P* < 0.05 versus diabetic group. Control, normal control group; DM, diabetic model group; Ad-GFP, Ad-SIK1, Met and ZQR shows groups treated with Ad-GFP, Ad-SIK1, metformin and ZQR, respectively.

The diabetic rats indicated typical diabetic symptoms polydipsia, polyuria and emaciation. These symptoms are related to the presence of hyperglycemia. Three rats died of hyperglycemia in the DM and Ad-GFP groups, while two rats died of hyperglycemia in the Ad-SIK1 and Met groups, respectively, and one rat died of hyperglycemia in the ZQR group.

To examine the effect of ZQR treatment on HFD/STZ-induced diabetic rats, we measured the final body weight and liver weight of rats. Injection of STZ dramatically decreased the weight of rats. Interestingly, the reduced body weight in the DM group was elevated by ZQR or metformin, as shown in Table 1. Parallel to the body weight change, the hepatic weight and the liver weight index (the ratio of liver to body weight) in the DM group were greater than those in the normal control group, while the liver weight and liver index of the DM group were markedly reduced by metformin or ZQR extract after 12 weeks of treatment. These findings show that ZQR supplementation could suppress hepatomegaly and attenuate the symptoms of diabetic rats.

**Table 1:** Effect of ZQR on body and liver weight in diabetic rats

Group	n	Body weight (g)	Liver weight(g)	Liver weight (%)
Control	8	509.3 ± 26.20	18.97 ± 4.33	3.71 ± 0.76
DM	5	366.7 ± 68.55***	28.52 ± 2.75***	7.29 ± 1.88**
Ad-GFP	5	373.1 ± 20.79**	27.88 ± 3.21***	7.14 ± 0.86**
Ad-SIK1	6	363.6 ± 51.18***	27.25 ± 4.11***	7.05 ± 1.17
Met	6	397.8 ± 58.41**	19.68 ± 2.93#	6.18 ± 1.90#
ZQR	7	387.4 ± 51.41***	21.51 ± 2.11#	6.22 ± 0.76#

# group(s) was significantly (*p* < 0.05) different from diabetic model group ** *P* < 0.01, *** *P* < 0.001 versus control group

### Measurement of metabolic parameters

The data are expressed as the mean ± SD. ^*^P < 0.05, ^**^P < 0.01, ^***^ *P* < 0.001 versus control group. ^#^*P* < 0.05, ^##^*P* < 0.05, ^###^*P* < 0.05 versus diabetic model group. Control, normal control group; DM, diabetic model group; Ad-GFP, Ad-SIK1, Met and ZQR shows groups treated with Ad-GFP, Ad-SIK1, metformin and ZQR, respectively.

Table 2 indicated that the blood glucose, TG and TC in the diabetic model group were drastically higher compared with the normal control group. Metformin or ZQR administration markedly decreased the FBG level in the Met and ZQR groups compared with the level in the DM group. Moreover, serum TG was markedly decreased but serum TC slightly was decreased in the Ad-SIK1, ZQR and Met groups compared to levels in the DM group. Consistent with a reduction in serum TG levels, the hepatic TG content was markedly reduced by treatment with ZQR or Ad-SIK1. Although ZQR alleviated the TC level of diabetic rats, there was no significant difference. Intriguingly, Ad-SIK1 treatment lowered blood glucose, but no significant difference was observed. Compared with the normal group, STZ induced hyperglycemia but led to decreased levels of serum insulin. The administration of ZQR and metformin caused a significant increase in the serum insulin levels compared with those in the DM group, but no significant difference was observed. Ad-SIK1 treatment showed no remarkable increase in insulin levels. These results show that ZQR administration improved the metabolic parameters in diabetic rats.

**Table 2:** Effect of ZQR on metabolic parameters in diabetic rats

Group	FBG (mmol/L)	TG (mmol/L)	TC (mmol/L)	Insulin (mU/L)	Hepatic TG (mmol/gprot)
Control	5.6 ± 0.9	0.84 ± 0.13	2.3 ± 0.63	42.51 ± 6.56	0.0628 ± 0.0050
DM	27.09 ± 2.69***	5.74 ± 1.69***	3.89 ± 1.93	26.26 ± 3.44***	0.196 ± 0.0276
Ad-GFP	26.99 ± 2.17***	5.30 ± 1.77***	3.89 ± 1.26	27.92 ± 6.11***	0.202 ± 0.1075
Ad-SIK1	25.71 ± 3.88***	1.68 ± 1.02###	2.31 ± 0.54	28.74 ± 5.63**	0.1051 ± 0.0157
Met	17.32 ± 4.45#	1.22 ± 0.31###	2.13 ± 0.5	36.83 ± 5.81#	0.1006 ± 0.0221#
ZQR	21.08 ± 6.26#	1.83 ± 0.79###	2.58 ± 0.65	35.03 ± 6.52#	0.091 ± 0.0262##

*P < 0.05, **P < 0.01, *** *P* < 0.001 versus control group

#*P* < 0.05, ##*P* < 0.05, ###*P* < 0.05 versus diabetic model group

### Histological examination of the liver and pancreas

**Fig. 1:**
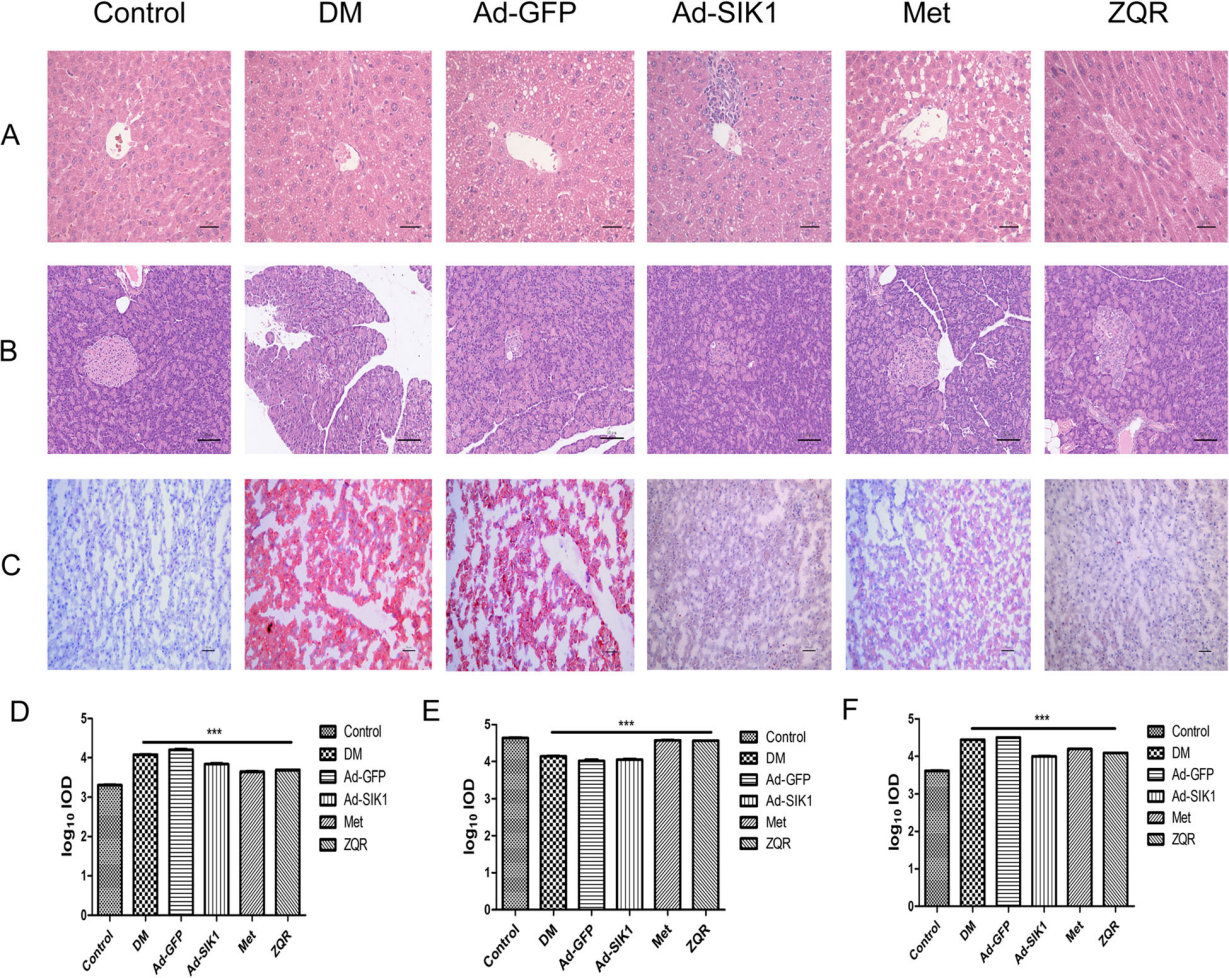
Effect of ZQR on the histology of the liver and pancreas in diabetic rats. **a** and (**c**) Representative photos of H&E or Oil red O staining from liver. **b** The pancreas was stained with HE (× 200) to observe islet morphology. **d** and (**f**) The area of lipid droplets. **e** The islet size. The scale bar is 50 μm

As shown in Fig. 1, parallel to the biochemical results, H&E or Oil red O staining results showed hepatic lipid deposition in the DM group, whereas less lipid deposition was observed in the liver of the ZQR and Met groups. Thus, the ZQR and Met groups had obviously decreased lipid deposition compared with that of the DM group, indicating that ZQR and Met could significantly improve steatosis. Additionally, complete pancreatic structure, normal islet shape and islet cell boundaries were observed in the control group (Fig. 1b). In contrast, the diabetic rats showed significant damage to the islet cells with smaller islets and fewer islet cells, and the histopathological changes in the Ad-GFP and Ad-SIK1 groups were similar to those in the DM group. However, rats in the Met and ZQR groups showed different degrees of repair of islet cell damage, and the number of islet cells and the morphology of islets were close to normal, indicating that the hypoglycemia effect of Met and ZQR was associated with repaired pancreatic tissue injury. These results demonstrated that ZQR improved the pathological changes of liver and played a protective role in diabetic rats.

### Effect of ZQR on the expression of proteins related to glucose metabolism in the rat liver

**Fig. 2:**
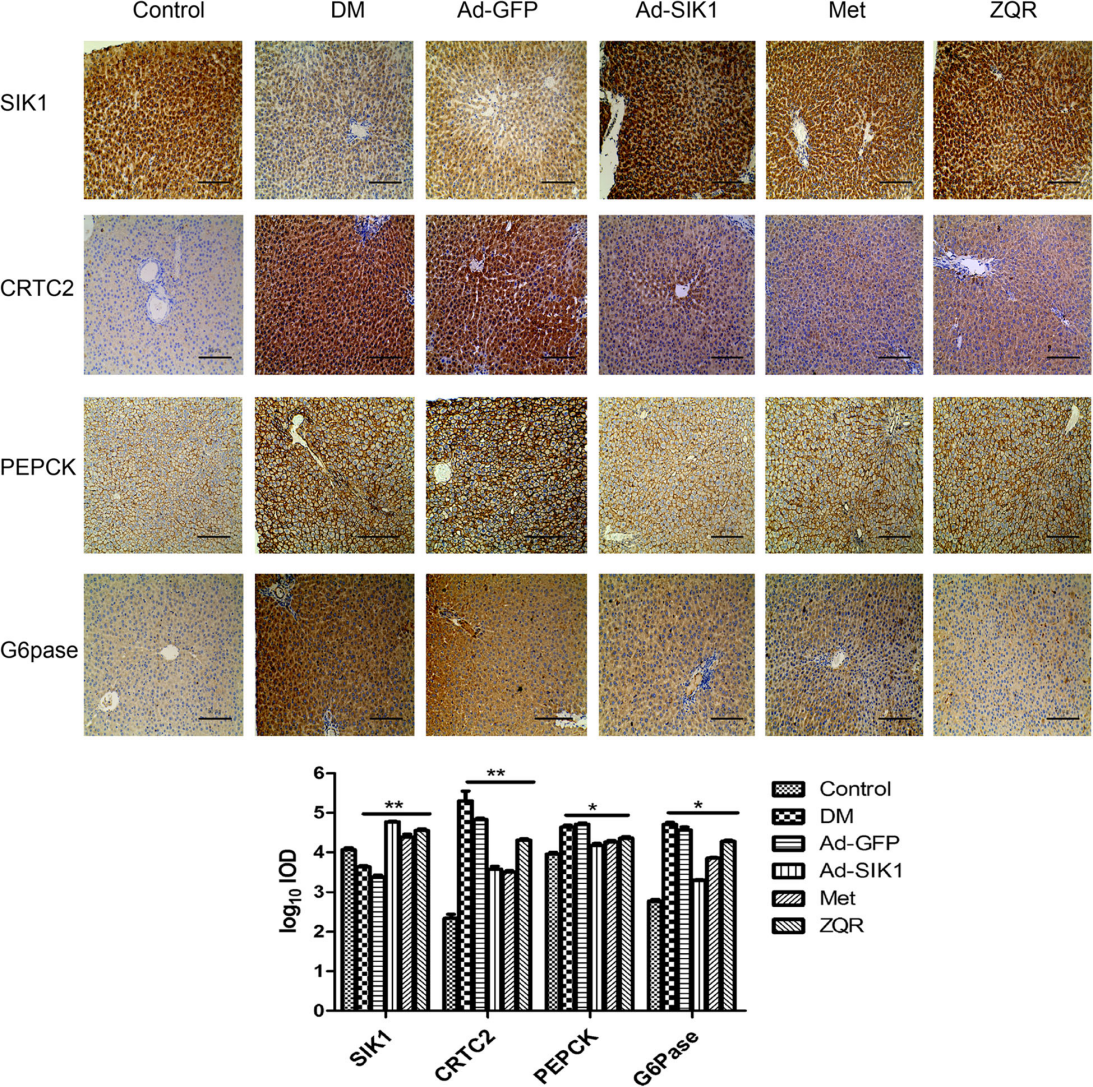
Effect of ZQR on the expression of proteins related to glucose metabolism in the diabetic rat liver (brown and yellow) (× 200). Immunohistochemical staining for proteins in the rat liver were illustrated. Staining for proteins was calculated. Scale: 50 μm. *P* < 0.05 (*), *P* < 0.01 (**)

As illustrated in Fig. 2, Diabetic rats expressed decreased SIK1, as well as increased CRTC2, PEPCK and G6Pase, compared with the normal control rats. Treating the diabetic rats with ZQR significantly increased the staining outcomes of SIK1 proteins, but obviously reduced those of CRTC2, PEPCK and G6Pase proteins.

### Effect of ZQR on immunohistochemical staining of insulin in the pancreas

**Fig. 3:**
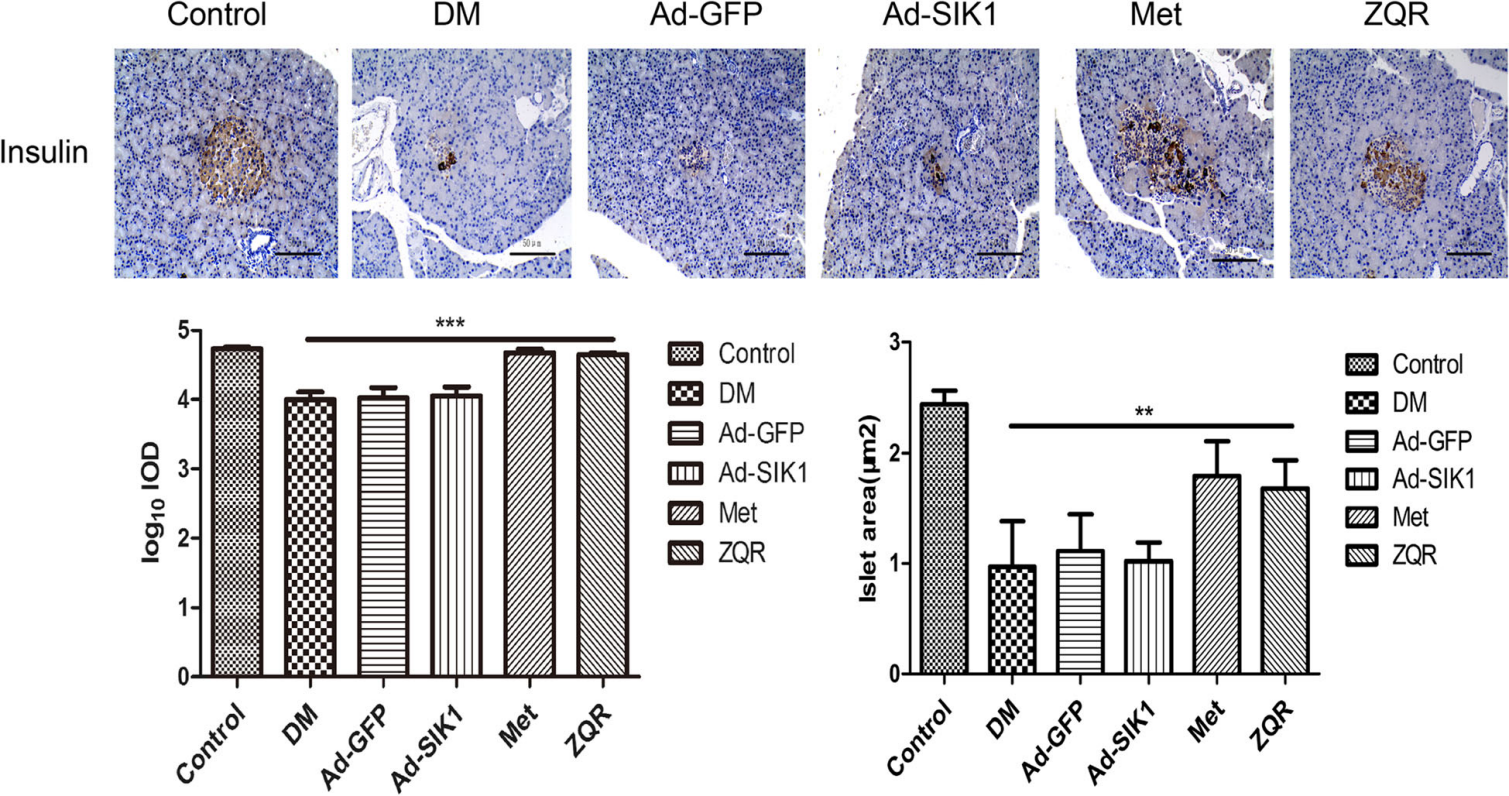
Immunohistochemical evaluation of pancreas (brown) (× 200). Scale: 50 μm

Figure 3 shows the number of β cells and the expression of insulin in islets. There was a significant decrease in the number of islet β cells as well as the expression of insulin in the pancreas of diabetic rats after STZ injection. ZQR or metformin administration inhibited the decrease in both the β cell number and insulin expression and restored them to near normal, while these significant changes were not observed in the Ad-SIK1 group. Thus, ZQR has a protective effect on the pancreas of diabetic rats by repairing islet damage.

### Effect of ZQR on the SIK1/CRTC2 pathway in rat liver

**Fig. 4:**
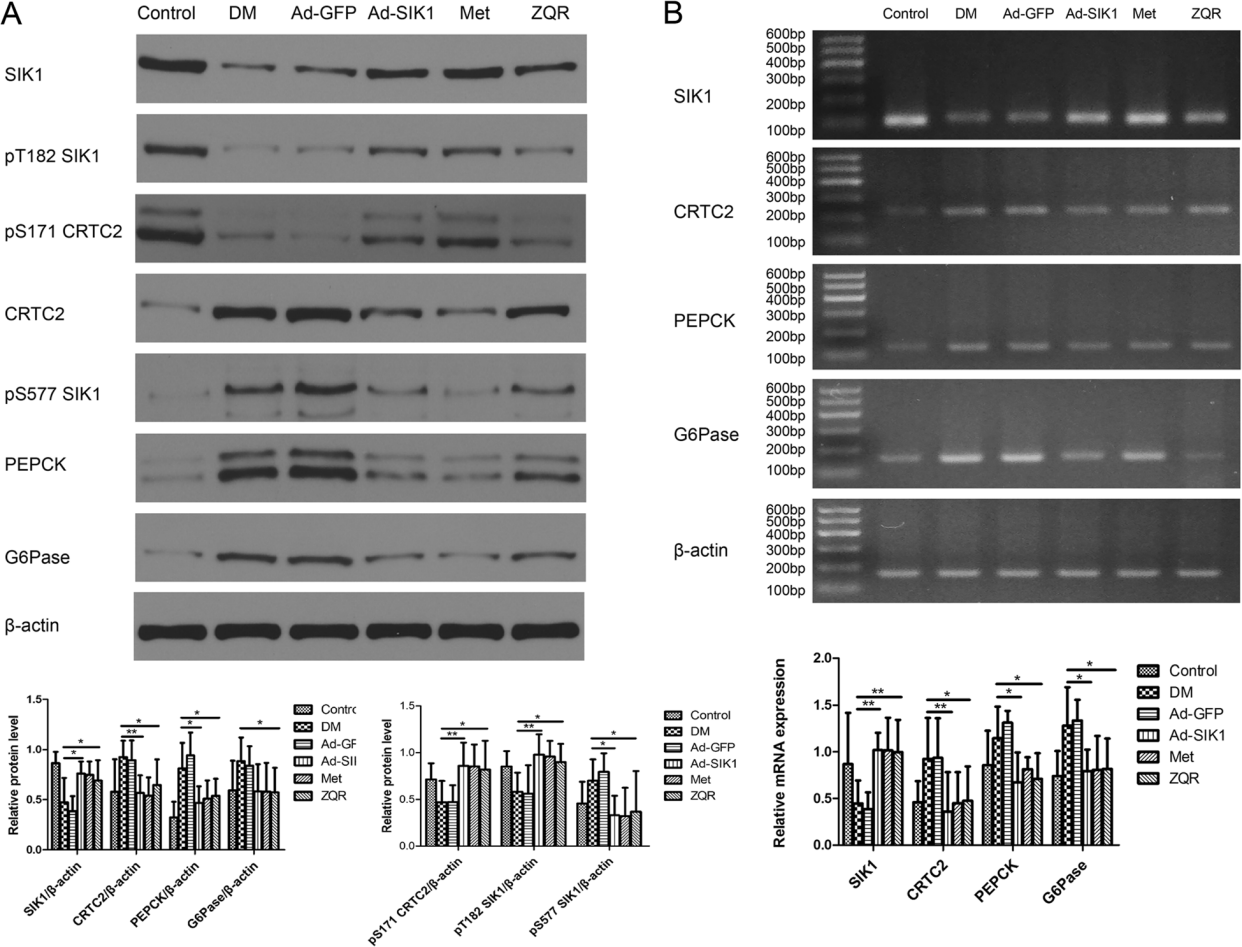
Effect of ZQR on the expression of genes related to glucose metabolism in the diabetic rat liver. Western blot analyses were shown in (**a**). RT-PCR analyses were shown in (**b**). Scale: 50 μm. *P* < 0.05 (*), *P* < 0.01 (**)

To further confirm the protective effect of ZQR on diabetes complicated by NAFLD, we examined the expression of glucose metabolism-associated genes in the liver of rats treated with ZQR. Diabetic rats produced less SIK1, SIK1-Thr 182 and CRTC2-Ser 171 protein, but more SIK1-Ser577, CRTC2, PEPCK and G6Pase protein than normal control rats in the liver, which likely led to the development of NAFLD. After ZQR administration, the expression of SIK1, SIK1-Thr 182 and CRTC2-Ser 171 was elevated and SIK1-Ser577, CRTC2, PEPCK and G6Pase were decreased (Fig. 4a, Additional file 3). Our findings were further investigated using RT-PCR analysis (Fig. 4b). Diabetic rats reduced the mRNA expression of SIK1 and increased that of CRTC2, PEPCK and G6Pase compared with normal control rats in the liver. ZQR administration obviously reversed the mRNA expression of SIK1, CRTC2, PEPCK and G6Pase in the liver of diabetic rats. These results suggest that ZQR could ameliorate hyperlipidemia in diabetic rats by regulating the SIK1/CRTC2 signaling pathway.

## Discussion

As a chronic metabolic disease, T2DM accounts for 90% of all diabetic mortality [31]. Therefore, it is urgent to find new and effective treatment methods. Some Chinese herbal prescriptions have recently attracted increasing attention due to their advantages of effectively reducing blood sugar and delaying complications [32, 33]. ZQR, a Chinese herbal formula, contains *Ligustrum lucidum* W.T.Aiton, *Eclipta prostrata* Lour and *Dioscorea oppositifolia* L.. Since ancient times, the herbal drugs of ZQR have been widely applied and have had a therapeutic effect on Xiao-ke, equivalent to diabetes mellitus in western medicine [26, 34, 35]. The herbal drugs of ZQR were verified using fingerprinting techniques [26], and its main ingredients were shown to reduce blood glucose and blood lipid levels in diabetic rats [36]. Our previous work indicated that ZQR ameliorated hyperglycemia, insulin resistance and fatty liver by suppressing FOXO1 in the liver of T2DM rats [27]. The present study further demonstrated its effects on hepatic glucose and lipid metabolism and its possible mechanisms.

In this work, we established a diabetic rat model with NAFLD induced by HFD and STZ. Consistent with previous reports [37, 38], the diabetic rats displayed obvious increases in serum glucose, TC and TG; fat droplet deposits in the liver; and severe pancreatic impairment, coupled with impairment of insulin secretion. After 12 weeks of treatment, ZQR treatment decreased FBG, TC and TG levels as well as lipid accumulation in the liver. Additionally, we observed an obvious increase in the serum insulin level in the ZQR group, indicating that ZQR might reduce blood sugar levels by promoting insulin secretion. Meanwhile, ZQR significantly rescued the morphological abnormalities in the liver and pancreas. There were similar therapeutic effects between the ZQR and Met groups. These results suggest that ZQR reduces hyperglycemia and hyperlipidemia, attenuated hepatic steatosis and improved insulin secretion in diabetic rats. The present results show that ZQR might be an effective drug for diabetes.

SIK1 plays an important role in gluconeogenesis [7, 22]. Koo et al. [22] demonstrated that SIK1 gene knockdown induced an increase in both fasting hyperglycemia and hepatic gluconeogenic gene expression in mice, which of them were decreased using overexpression of SIK1 in db/db mice. Moreover, Hepatic SIK1 activity was reduced in db/db mice [7]. These findings suggest that a decrease in the expression of SIK1 may be helpful to the disorders of glucolipid metabolism in diabetes, and certainly a method to overexpress SIK1 could lead to an efficient therapy for T2DM. To test this idea, high expression of SIK1 was induced in diabetic rats by tail vein injection of a recombinant adenoviral vector. As expected, Ad-SIK1 administration significantly inhibited hepatic gluconeogenesis and steatosis in diabetic rats. More importantly, Ad-SIK1 treatment lowered FBG, serum TC and TG levels and the hepatic TG content. These results suggest that SIK1 significantly regulates hepatic gluconeogenesis and the synthesis of lipids in vivo. Meanwhile, the hepatic expression of SIK1 was sinificantly reduced in diabetic rats. However, ZQR treatment significantly upregulated the mRNA and protein expression of SIK1 in the liver of diabetic rats. In addition, ZQR treatment drastically decreased serum glucose, TG levels and hepatic TG content. These results suggest that upregulation of SIK1 through ZQR may contribute to decreasing hyperglycemia, ameliorating lipid profiles and attenuating fatty liver. It has been reported that phosphorylation of SIK1 at Thr182 is essential for sustained activity of SIK1 [13, 39], and Thr-182 phosphorylation of SIK1 causes SIK1 kinase to change from inactivation to activation [12]. Our previous works have shown that high glucose levels significantly reduced the level of pT182 SIK1 in HBZY-1 and HepG2 cells [14, 30]. Consistent with these results, this in vivo study demonstrated that the phospho-Thr182 and expression of SIK1 was reduced in the liver of diabetic rats, suggesting that SIK1 activity is inhibited in diabetic states. Interestingly, similar to Ad-SIK1, ZQR treatment led to an evident increase in the level of Thr-182 phosphorylation, suggesting that ZQR might enhance the activity of SIK1 in the liver of diabetic rats. In addition, the distribution of SIK1 in cells is highly related to its function. Katoh et al. [12] reported that Ser577 phosphorylation of SIK1 caused the cytoplasmic localization of SIK1 and subsequently led to a decrease in the transcriptional regulation activity of SIK1. This effect prompted us to investigate the phosphorylation of SIK1 at Ser577 in the liver of diabetic rats. We found that the phospho-Ser577 was increased in the DM group, whereas ZQR administration significantly reduced its level of phosphorylation.

An unusual increase in hepatic glucose production via gluconeogenesis is an important reason leading to hyperglycemia in T2DM. Lin et al. [15] demonstrated that excessive hepatic glucose output in T2DM mainly resulted from continuous gluconeogenesis. The rate of gluconeogenesis mainly depends on gluconeogenic enzymes such as PEPCK and G6Pase. Berdeaux [7] demonstrated that the phosphorylation/dephosphorylation of CRTC2 had a physiological effect on hepatic gluconeogenesis. Adenovirus-expressed CRTC2 (Ad-CRTC2) promoted glucose production in primary hepatocytes. Moreover, Ad-CRTC2 promoted fasting hyperglycemia in vivo [22]. Mice with a CRTC2 defect showed hypoglycemia and maintained better insulin sensitivity when fed a HFD, and the mRNA expression of gluconeogenic genes was significantly decreased in the liver [22]. Interestingly, CRTC2 knock down reduced hepatic gluconeogenesis and triglyceride and improved insulin sensitivity in animal models of T2DM and insulin resistance [20]. Circulating insulin, triglyceride and cholesterol concentrations were down-regulated, whereas whole body insulin sensitivity was increased in CRTC2 knockout mice [21]. Persistent activation of CRTC2 in the liver was sufficient to promote hepatic gluconeogenesis, insulin resistance and steatosis [40]. In addition, activation of CRTC genes inhibited lipolysis, which would prevent energy consumption, in turn driving obesity, while the reduction in hepatic CRTC2 expression activated lipolysis, which contributed to the hydrolysis of lipids [41]. Therefore, these results suggest that CRTC2 not only regulates hepatic gluconeogenesis but also contributes to the development of insulin resistance and steatosis in part through its effects on hepatic gluconeogenesis. As described in detail previously [22], serine 171 was the main phosphorylation site that mediated CRTC2 activity, and SIK1 inhibited CREB activity by phosphorylating CRTC2 at Ser171 to suppress hepatic gluconeogenesis. This study showed that the expression of CRTC2, PEPCK and G6Pase was obviously upregulated, whereas pS171 CRTC2 was markedly reduced in the DM group compared to levels in the control group. ZQR treatment markedly increased the expression of SIK1 in the liver of diabetic rats, while those of CRTC2, PEPCK and G6Pase were significantly reduced by ZQR, thus leading to a reduction in blood glucose, serum lipid levels and hepatic TG. Our results were further confirmed using histological and immunofluorescence staining. The decreased mRNA and protein expression of CRTC2 was accompanied by elevated pS171 CRTC2 in the ZQR group compared with that in the DM group, indicating the importance of Ser 171 for the activity of CRTC2. These findings indicate that hepatic gluconeogenesis and steatosis were inhibited by ZQR, at least partially via the SIK1/CRTC2 pathway in diabetic rats.

In patients with T2DM, hyperglycemia is primarily due to a decreased ability of insulin to inhibit hepatic glucose production and the impairment of glucose uptake by insulin-sensitive tissues. Rakieten et al. [42] reported that insulinopenia was one of the important features of STZ-induced diabetic rodents, which resulted from severe damage to β cells in the pancreas. In the present study, a significant reduction in serum insulin levels was observed with a consequent increase in blood glucose levels in the diabetic rats. ZQR treatment for 12 weeks caused a remarkable increase in serum insulin levels, indicating that the hypoglycemic effect of ZQR might be due to its effect on the pancreas by promoting insulin secretion and/or inducing β cell regeneration. To confirm this hypothesis, we further performed an immunohistochemical examination of the pancreatic tissue. Notably, ZQR and metformin significantly elevated the areas of insulin-positive cells compared to those of the DM group, thus resulting in an increase in serum insulin levels followed by a decrease in blood glucose levels. There are several reasons for the above results. First, the biologically active ingredients of ZQR might work by promoting insulin secretion from the remnant β cells. Second, ingredients existing in ZQR might increase the sensitivity of insulin, thereby suppressing hepatic gluconeogenesis. Third, the decrease in liver and serum TG levels might help to increase insulin sensitivity due to a decrease in lipotoxicity and/or glucotoxicity. Under physiological conditions, insulin inhibits hepatic gluconeogenesis and glycogenolysis by regulating gluconeogenic genes such as PEPCK and G6Pase and their expression [43].

Metformin has been widely used to treat T2DM due to its effects in deterring hepatic gluconeogenesis and improving peripheral insulin sensitivity [22]. In this study, metformin was also shown to upregulate SIK1 expression and suppress the expression of hepatic glucogenic and lipogeic genes in diabetic rats. However, metformin was better than ZQR at attenuating hepatic gluconeogenesis and lipogenesis.

## Conclusion

**Fig. 5:**
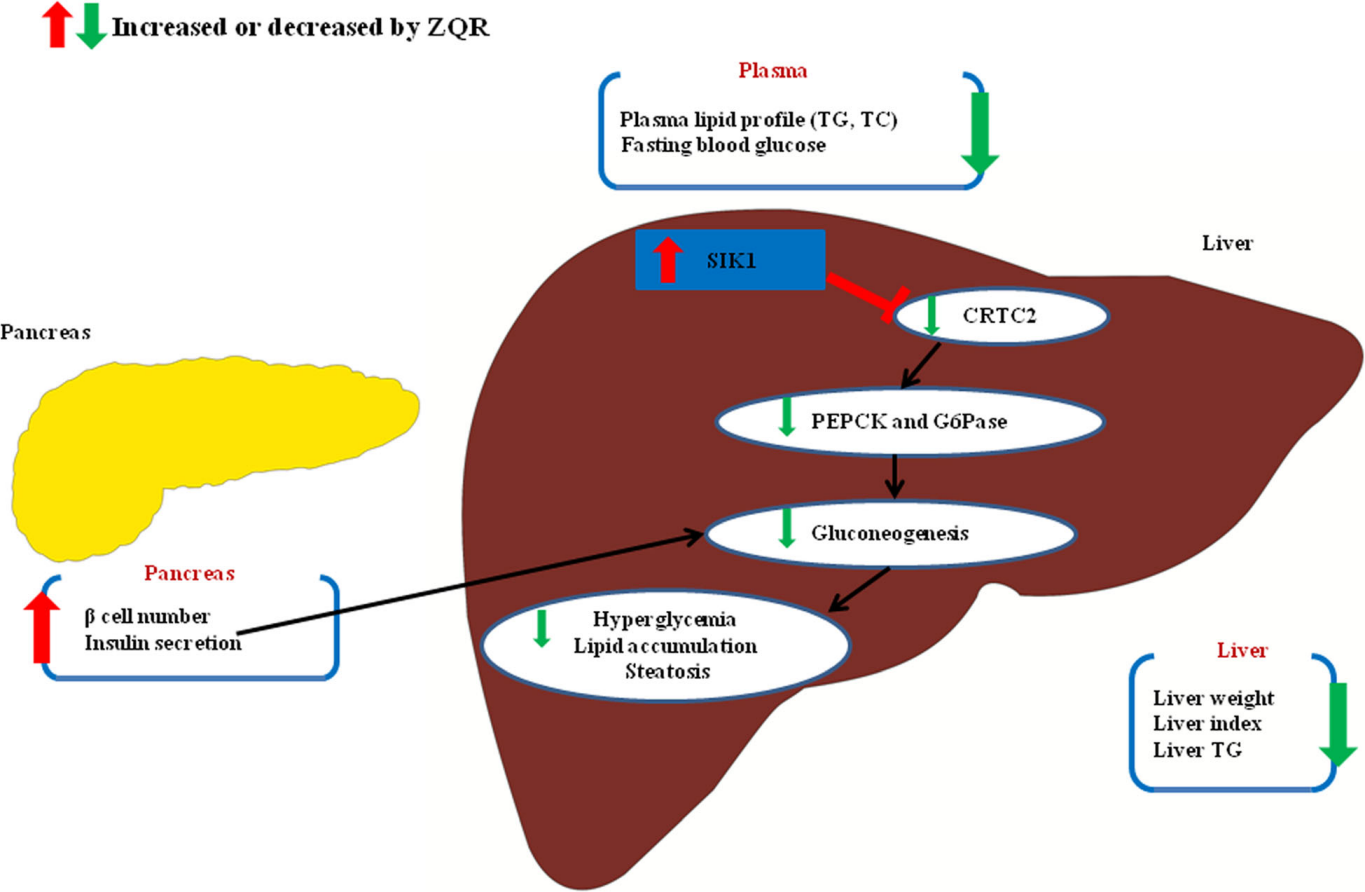
Schematic diagram of the mechanism of ZQR against NAFLD. ZQR alleviates NAFLD through regulating glucose and lipid metabolism via SIK1/CRTC2 signaling pathway

In conclusion, the present study revealed that SIK1 inhibits hepatic gluconeogenesis and steatosis and that proper regulation of CRTC2 activity by SIK1 kinase is essential for suppressing abnormal hepatic glucose production and lipid storage. ZQR significantly upregulates SIK1 and downregulates CRTC2 as well as glucogenic genes in the liver of diabetic rats. ZQR drastically decreases blood glucose and lipid levels, improves insulin secretion and ameliorates histopathological changes in the liver and pancreas of diabetic rats. The mechanism of action of ZQR may be closely associated with its inhibition of hepatic gluconeogenesis and lipid accumulation through activating the SIK1/CRTC2 pathway. Taken together, this study suggests that enhancing SIK1 activity with ZQR would be expected to decrease hyperglycemia and hyperlipidemia as well as hepatic lipid storage in patients with diabetes with NAFLD. Figure 5 shows the role of ZQR in amelioration of NAFLD.

## Supplementary information

**Additional file 1.** ARRIVE checklist.

**Additional file 2: Table S1.** Sequences of primers used for PCR analysis.

**Additional file 3.** Uncropped western blots.

## Data Availability

The supporting materials can be obtained upon request via email to the corresponding author.

## Abbreviations

ACTH: Adrenocorticotropic hormone
Ad-GFP: Adenovirus-green fluorescent protein
Ad-SIK1: Adenovirus-Salt induced kinase 1
AMPK: AMP-activated protein kinase
CREB: cAMP response element binding protein
CRTC2: CREB-regulated transcription co-activator 2
DM: Diabetes mellitus
FBG: Fasting blood glucose
G6Pase: Glucose-6-phosphatase
HFD: High fat diet
IOD: Integrated optical density
LKB1: Serine/threonine kinase 11
Met: metformin
NAFLD: Nonalcoholic fatty liver disease
OD: Optical density
PEPCK: Phosphoenolpyruvate carboxykinase
PFU: Plaque forming units
PGC-1α: Peroxisome proliferator-activated receptor gamma coactivator 1-alpha
PKA: Protein kinase A
RT-PCR: Reverse transcription-polymerase chain reaction
SIK1: Salt induced kinase 1
STZ: Streptozotocin
T2DM: Type 2 diabetes mellitus
TC: Total cholesterol
TG: Triglycerides
ZQR: Zhenqing recipe
