# Supplementary material for: Zhenqing recipe attenuates non-alcoholic fatty liver disease by regulating the SIK1/CRTC2 signaling in experimental diabetic rats
Source: BMC Complement Med Ther. 2020 Jan 31;20:27. doi: 10.1186/s12906-019-2811-2 (PMC7076741; doi:10.1186/s12906-019-2811-2)
Supplement: Supplementary file 2 — Additional file 2: Table S1. Sequences of primers used for PCR analysis. [file 12906_2019_2811_MOESM2_ESM.docx]

**Supplementary Table 1.** Sequences of primers used for PCR analysis.

| **Gene** | **Forward Primer(5´-3´)** | **Reverse Primer(5´-3´)** |
| --- | --- | --- |

| SIK1 | GGCGACTACAACGAGCAGGT | | | GCAGGAGGTAGTAAATAGCCGC | |
| --- | --- | --- | --- | --- | --- |
| CRTC2 | AGGCCTGCTTAGTACAGCCCT | | | GGCCACTCACTGATCCCTCA | |
| PEPCK | ACCAGTGATGGCGGTGTGTA | | | AAAGCGAGAGTTTGGATGCG | |
| G6pase | TTCTATGTCCTCTTTCCCATCTG | | | CACGGAGCTGTTGCTGTAATAG | |
| SREBP-1c | GCGCTACCGTTCCTCTATCAA | | | CTTCGCAGGGTCAGGTTCTC | |
| FAS | ATGGCTGTCCTGCCTCTGGT | | | ACGCTCCTCTTCAACTCCAAA | |
| ACC | GAGATTTCACTGTGGCTTCGC | | TAATTGTTGTTGTTTGCTCCTCC | | |
| β-actin | | CGTTGACATCCGTAAAGACCTC | | | TAGGAGCCAGGGCAGTAATCT |
