# Supplementary figures and images for: Zhenqing recipe attenuates non-alcoholic fatty liver disease by regulating the SIK1/CRTC2 signaling in experimental diabetic rats
Source: BMC Complement Med Ther. 2020 Jan 31;20:27. doi: 10.1186/s12906-019-2811-2 (PMC7076741; doi:10.1186/s12906-019-2811-2)

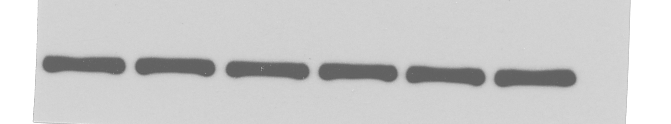

Supplement: Supplementary file 3 — Additional file 3. Uncropped western blots. [file 12906_2019_2811_MOESM3_ESM.zip › ACTINR5.tif]

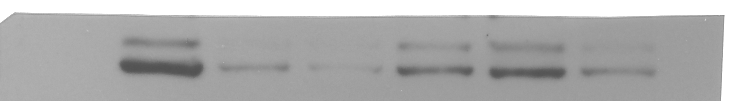

Supplement: Supplementary file 3 — Additional file 3. Uncropped western blots. [file 12906_2019_2811_MOESM3_ESM.zip › CRTC2(s171)R5.tif]

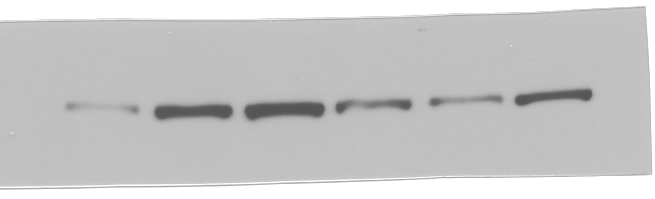

Supplement: Supplementary file 3 — Additional file 3. Uncropped western blots. [file 12906_2019_2811_MOESM3_ESM.zip › CRTC2R5.tif]

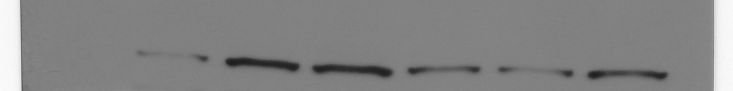

Supplement: Supplementary file 3 — Additional file 3. Uncropped western blots. [file 12906_2019_2811_MOESM3_ESM.zip › g6pR5.tif]

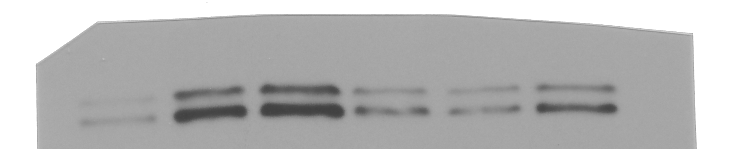

Supplement: Supplementary file 3 — Additional file 3. Uncropped western blots. [file 12906_2019_2811_MOESM3_ESM.zip › PEPCKR5.tif]

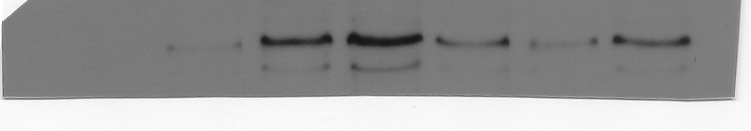

Supplement: Supplementary file 3 — Additional file 3. Uncropped western blots. [file 12906_2019_2811_MOESM3_ESM.zip › renamed_b51f0R5.tif]

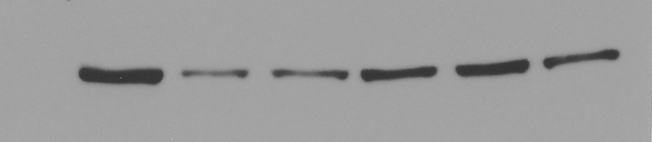

Supplement: Supplementary file 3 — Additional file 3. Uncropped western blots. [file 12906_2019_2811_MOESM3_ESM.zip › SIK1R5.tif]

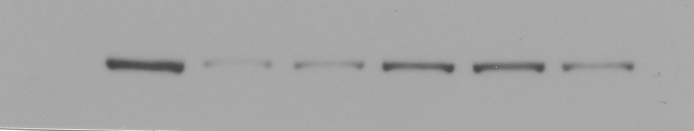

Supplement: Supplementary file 3 — Additional file 3. Uncropped western blots. [file 12906_2019_2811_MOESM3_ESM.zip › T182 SIK1R5.tif]
